# Dynamic Supramolecular Ruthenium‐Based Gels Responsive to Visible/NIR Light and Heat

**DOI:** 10.1002/chem.201902088

**Published:** 2019-07-03

**Authors:** Ian Teasdale, Sabrina Theis, Aitziber Iturmendi, Moritz Strobel, Sabine Hild, Jaroslaw Jacak, Philipp Mayrhofer, Uwe Monkowius

**Affiliations:** ^1^ Institute of Polymer Chemistry Johannes Kepler University Linz Altenberger Straße 69 4040 Linz Austria; ^2^ Institute of Inorganic Chemistry Johannes Kepler University Linz Altenberger Straße 69 4040 Linz Austria; ^3^ Institute of Polymer Science Johannes Kepler University Linz Altenberger Straße 69 4040 Linz Austria; ^4^ School of Medical Engineering and Applied Social Sciences University of Applied Sciences Upper Austria Garnisonstraße 21 4020 Linz Austria; ^5^ School of Education Johannes Kepler University Linz Altenberger Straße 69 4040 Linz Austria

**Keywords:** metallopolymers, multiphoton absorption, photosensitive gels, ruthenium, self-healing

## Abstract

A simple supramolecular crosslinked gel is reported with a photosensitive ruthenium bipyridine complex functioning as a crosslinker and poly(4‐vinylpyridine) (P4VP) as a macromolecular ligand. Irradiation of the organogels in H_2_O/MeOH with visible and NIR light (in a multiphoton process) leads to cleavage of pyridine moieties from the ruthenium complex breaking the cross‐links and causing degelation and hence solubilization of the P4VP chains. Real‐time (RT) photorheology experiments of thin films showed a rapid degelation in several seconds, whereas larger bulk samples could also be photocleaved. Furthermore, the gels could be reformed or healed by simple heating of the system and restoration of the metal–ligand crosslinks. The relatively simple dynamic system with a high sensitivity towards light in the visible and NIR region make them interesting positive photoresists for nano/micropatterning applications, as was demonstrated by writing, erasing, and rewriting of the gels by single‐ and multiphoton lithography.

The field of supramolecular polymers aims to use directional intermolecular forces^1^ to prepare dynamic materials. These so called “dynamers” are designed to undergo reorganization by reversible chemical reactions under the influence of external factors.^2^ The dynamicity can be incorporated either by reversible covalent or noncovalent bonding, such as defined hydrogen‐bonding moieties, or alternatively by ionic bonding or coordinating metal bonds.^2^ Metallopolymers can offer unique properties and a number of supramolecular systems have been developed based on dynamic ligand–metal interactions.^3^ Such materials have been investigated in a wide field of applications, for example the transduction of mechanical force into chemical reactions,^4^ as well as in dynamic self‐healing^5^ and optically healable materials.^6^ Also a number of ruthenium‐containing metallopolymers have been reported, with a particular focus on their use as stimuli‐responsive polymers.^7^ Meanwhile, photocleavable polymers, that is polymers which cleave or degrade in response to irradiation with certain wavelengths of light have been recently developed.^8^ Incorporation of known photocleavable protecting groups, also sometimes referred to as photocages,^9^ into macromolecular systems is one route to achieve this goal. Examples include the incorporation of coumarin^10^ or *o*‐nitrobenzyl^11^ groups into polymeric materials^10^, ^12^ to achieve selective photodegradation.

While materials responsive to UV‐light have long been established, there has been a recent shift in the field towards the use of photochemical processes, which respond in the visible and NIR region11a, 13 due to the poor penetration of high‐energy UV‐light as well as its incompatibility with biological environments.^14^ Also light‐sensitive ruthenium‐based complexes have been shown to be responsive to light with long wavelengths,^15^ for example, for photodynamic therapy with deep‐tissue penetration. Various complexes based on a *cis*‐[Ru^II^(bpy)_2_(L_1_)(L_2_)]^2+^ structure are established as photocages^16^ and show a rapid cleavage of ligands upon excitation with visible light, as well as a two‐photon process in the NIR region. Furthermore, such complexes have recently been incorporated into hydrogels to achieve degelation and enable microphotopatterning^17^ and the light‐triggered release of enzymes.^18^ Herein, we report the use of ruthenium bipyridine complexes as a crosslinker for the polymer poly(4‐vinylpyridine) (P4VP), which acts as a macromolecular ligand, binding to the ruthenium centers and hence cross‐linking the polymers. The procedure of the gel preparation was identical for all gels, and just the concentration of the ruthenium complex was varied. Briefly, poly(4‐vinylpyridine) (P4VP; *M*_W_≈60 000 g mol^−1^) was crosslinked with [Ru(bpy)_2_Cl_2_] through complexation with the polymer‐bound pyridine ligands in a water/methanol (*v*/*v*=2:3) mixture at 80 °C for 16 h (Scheme 1) upon which gelation occurred (see the Supporting Information for full experimental details). The ligand exchange at the ruthenium atom with P4VP can be observed by UV/Vis spectroscopy (Figure 1), shown as an example for the gel with 5 mol % of the ruthenium complex. A thin layer of the gel (1 mm) was applied between two glass slides and measured in the UV/Vis spectrometer. For comparison, the spectrum of [Ru(bpy)_2_Cl_2_] is included in Figure 1 with its characteristic MLCT transition observed at 491 nm. The complexation with the pyridine moieties to the ruthenium atom shifts the peak maximum to 461 nm with a shoulder at ≈435 nm. The observed shift is typical for the MLCT peak of comparable ruthenium–pyridine complexes.^17^, ^19^ Above ≈600 nm no significant absorption is observed (see Figure S4, Supporting Information).

**Scheme 1:**
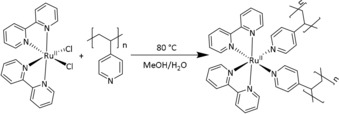
Reaction scheme for the synthesis of [Ru(bpy)_2_Cl_2_] and poly(4‐vinylpyridine).

**Figure 1:**
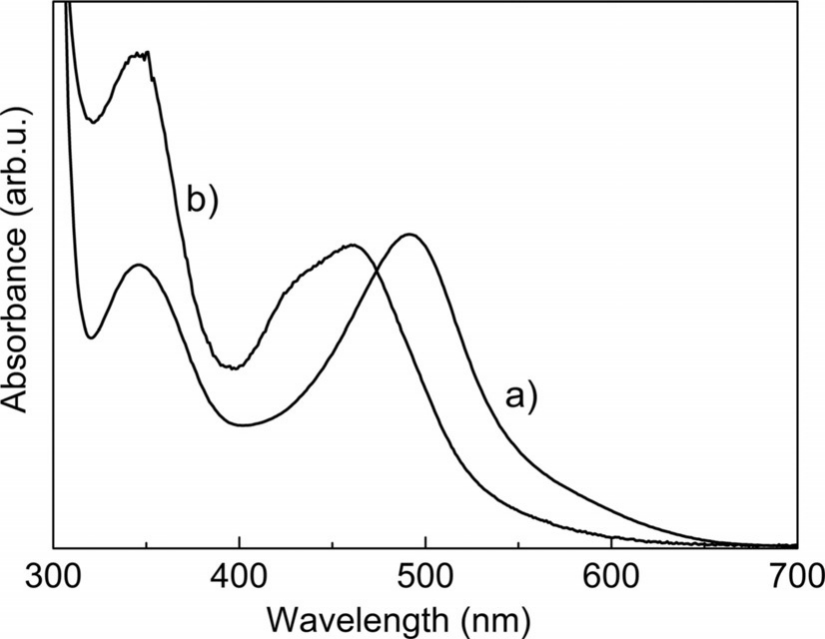
Absorption spectra of (a) [Ru(bpy)_2_Cl_2_] in water and (b) the ruthenium containing P4VP gel in water/methanol (5 mol % Ru).

To compare different crosslinker ratios, bulk gels with 5, 10, 15, and 20 mol % of [Ru(bpy)_2_Cl_2_] per pyridine unit were prepared (see the Supporting Information). Accordingly, with higher ruthenium ratios, the color of the obtained gel becomes darker and the gel more brittle. Additionally, the crosslinking ratio also impacts the gelation time. P4VP gels with 20 mol % of [Ru(bpy)_2_Cl_2_] are formed in about 15 minutes while standing at room temperature and gels with 5 mol % require up to 60 minutes for complete gelation.

All obtained gels remain solid in the dark for at least >12 months. The sensitivity to light was investigated by photorheology for polymers with 5 % ruthenium cross‐linker content (Figure 2). Real‐time‐(RT)‐photorheology experiments in which thin films are exposed to a light source were directly measured on the rheometer plates.^20^ The storage modulus G′ and the loss modulus G′′ of the gels were measured in oscillation mode (1 % deformation, 10 rad s^−1^, for further details and explanations see the Supporting Information). Upon irradiation (365 nm, 25 000 mW cm^−2^) an immediate and rapid decrease in moduli was observed, indicative of a de‐crosslinking of the gel^21^ through cleavage of the pyridine moiety of the P4VP from the complex.

**Figure 2:**
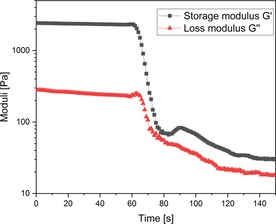
Polymer with 5 mol % ruthenium cross‐linker (85.9 mg in 2.5 mL water/methanol (*v*/*v*=2:3)). Light was switched on after 60 s. The significant decrease in moduli represents a reduced resistance to deformation, indicating a liquefaction of the gel caused by de‐crosslinking upon irradiation.

Degelation of the bulk samples was also investigated. Upon irradiation of the P4VP gel in a 10 mL pyrex culture tube with visible light >395 nm the gel becomes liquid. The kinetics differ from the RT‐rheology experiments due to the different light source used and different thickness and hence penetrability of the samples. As expected, the degree of crosslinking ratio also impacts the cleavage time due to the increased number of crosslinks but predominantly due to the increased absorbance of the incident light by the ruthenium chromophore. While the P4VP with 5 mol % Ru crosslinker becomes dissolved within 10 minutes, the sample with 15 mol % requires up to 60 minutes until the reaction mixture becomes liquid (see Figures S1–S3, Supporting Information). Meanwhile for the P4VP gel with 20 mol % of [Ru(bpy)_2_Cl_2_] no degelation was observed, presumably due to higher absorption of light and hence the lower penetration of light into the system. The cleavage of the ligand can also be followed by UV/Vis spectroscopy (Figure S4, Supporting Information). Since only one of the pyridine ligands is cleaved in water^17^, ^22^ the change in the absorption spectra is indicated through a slight shift of the MLCT band.

It was postulated that since the gelation reaction is initiated by an exchange of H_2_O ligands in the ruthenium complex with pyridine ligands,^17^ and since [Ru(bpy)_2_(H_2_O)(py‐P)]^2+^ (py‐P=one pyridine group of P4VP) is known to be the product of the photoreaction, then the gelation reaction would be thermally reversible (Scheme 2). Simple heating of the sample in the (RT)‐photorheology experiments indeed showed an increase in moduli (Figure S5, Supporting Information), although heating of thin films caused some solvent evaporation, which may interfere with the read‐out. The process was thus investigated on bulk samples with a rotational viscometer in a pyrex culture tube. The viscosity of the ≈15 % P4VP [Ru(bpy)_2_Cl_2_]^2+^ mixture increased to a maximum of 12 800 mPas upon heating for 16 hours at 80 °C (Figure 3). Upon irradiation with visible light >395 nm, the value decreases to near its initial value of around 320 mPas, suggesting a near complete degelation and hence solubilization of the P4VP chains.

**Scheme 2:**
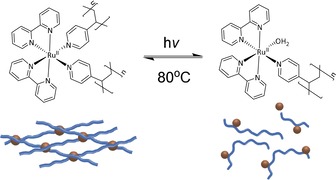
Dynamic reversible gels formed by binding of ruthenium to the P4VP macromolecular ligand.

**Figure 3:**
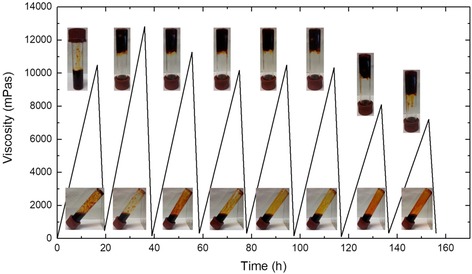
Rotational viscosity measurements of the ruthenium cross‐linked P4VP (≈15 % Ru). The gelation/de‐gelation cycle is as follows: Complexation by heating at 80 °C, gelation at r.t., measurement of viscosity, irradiation at *λ*>395 nm, measurement of viscosity.

Interestingly the reversible gelation/degelation process could be repeated with a number of cycles, as shown for the gel with ≈15 % Ru in Figure 3 and Figure S5, Supporting Information. The maximum viscosity of the gel appears to decrease with increasing number of cycles, suggesting a reduced extent of crosslinking as the system is reheated. It should, however, be stressed, that the viscosity experiments had to be carried out under ambient conditions. After 8 cycles gelation could not be achieved for this cross‐linker concentration. This is presumably due to some oxidation of the ruthenium centres to Ru^III^ over time. It is well known that Ru^II^–bpy complexes are efficient photoreductants.^23^ The formed Ru^III^ complexes are in general less prone to ligand exchange reactions and to our knowledge no analogous photosubstitution has been reported for Ru^III^–bpy complexes. Hence, the decreasing number of reactive ruthenium(II) centres available for crosslinking leads to a loss of gelation ability.

Since it was anticipated that the photocleavage is locally restricted, a gel containing test tube was covered with aluminum foil leaving a strip of 1 cm uncovered. The tube was then exposed to visible light leading to the degelation and solubilization of the P4VP chains (Figure S7, Supporting Information). Upon subsequent immersion of the test tube in a water bath at 80 °C, a complete gel was obtained again, suggesting a potential use of the polymers as healable materials.

Furthermore, to demonstrate the potential for micropatterning, logos were written into gel portions (Figure 4) containing 5 and 15 mol % of ruthenium crosslinker. These polymer samples were enclosed between two glass slides and properly sealed. The micropatterning could be achieved both by focused NIR‐laser light at 1028 nm (2 mW 15 % gel, 20 mW 5 % gel) in a multiphoton process,^24^, ^25^ as well as by single‐photon laser at single‐photon lithography (1PL) at 514 nm (2 μW 15 % gel). To verify the micropatterning, images were acquired directly after writing with the same setup using an infinity corrected NIR air microscopy objective (50×/0.42). For imaging we used either an industrial camera for white light images or a single photon avalanche diode for backscattered images (see Figure S8, Supporting Information). Upon smoothly heating the gel samples on a hot plate at 60 °C for one hour, the logos were observed to disappear. Subsequently, the same area of the gel was rewritten using the same MPL and 1PL conditions. All acquired images were contrast enhanced.

**Figure 4:**
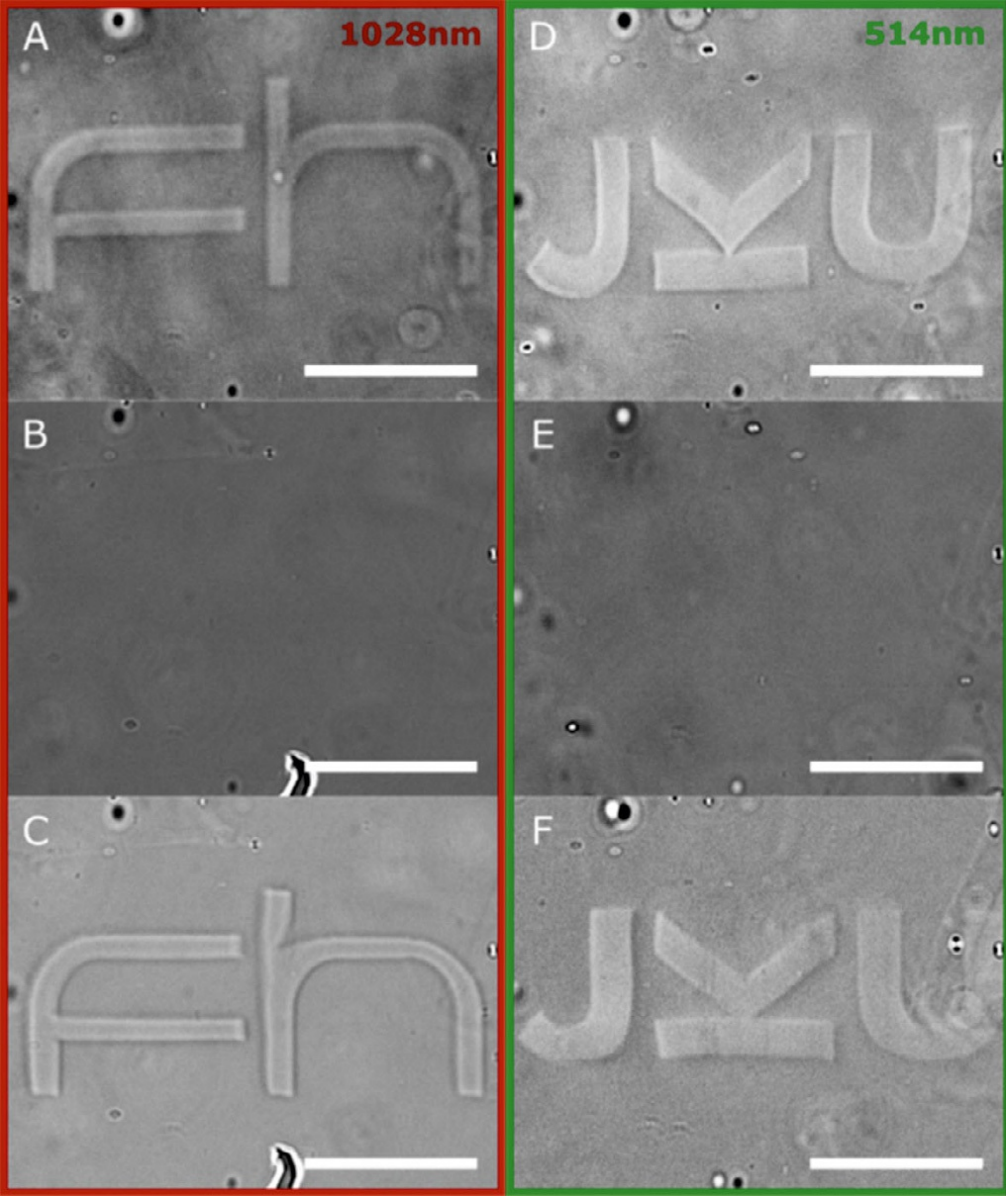
Contrasted white light images showing the writing, erasing and rewriting of the gels. (A) The 15 mol % hydrogel after writing the FH logo by multiphoton photon lithography (MPL) at 1028 nm. (B) After thermal healing treatment. (C) Rewriting the same area by MPL. (d–F) The same process is repeated with single photon lithography (1PL) at 514 nm. All scale bars are 50 μm.

In summary, a simple crosslinked gel was prepared based on P4VP as a macromolecular ligand cross‐linked with [Ru(bpy)_2_Cl_2_] complexes. Photocleavage of the metal–ligand bond upon irradiation broke the linkage between the macromolecules leading to degelation and hence solubilisation of the PV4P chains. RT‐rheology experiments of thin films showed a rapid degelation in several seconds while larger bulk samples could also be photocleaved in several minutes, depending on the concentration of ruthenium and penetrability of light into the gels. Furthermore the system was shown to be reversible with the gel being reformed upon heating, a process which could be cycled a number of times. The gels were prepared by simple mixing and heating and the properties could be tuned by the ratio of crosslinker to polymer. The relative simplicity of the system combined with the high sensitivity of the systems to light in the visible and NIR region make them highly interesting novel materials for lithographic applications. In this context micropatterning of the gels was demonstrated by multiphoton and single photon lithography at 1028 and 514 nm, respectively and it was shown that the gels could be positive photoresist erased, healed, and rewritten on the same region of the gel.

## Conflict of interest

The authors declare no conflict of interest.

## Supporting information

As a service to our authors and readers, this journal provides supporting information supplied by the authors. Such materials are peer reviewed and may be re‐organized for online delivery, but are not copy‐edited or typeset. Technical support issues arising from supporting information (other than missing files) should be addressed to the authors.

Supplementary

## References

[1] J. M. Lehn, Supramolecular chemistry, Concepts and perspectives, Wiley-VCH, Weinheim, **1995**.

[2] N. Roy , B. Bruchmann , J.-M. Lehn , Chem. Soc. Rev. 2015, 44, 3786–3807. 25940832 10.1039/c5cs00194c

[3a] A. Winter , U. S. Schubert , Chem. Soc. Rev. 2016, 45, 5311–5357; 27218823 10.1039/c6cs00182c

[3b] G. R. Whittell , M. D. Hager , U. S. Schubert , I. Manners , Nat. Mater. 2011, 10, 176–188. 21336298 10.1038/nmat2966

[4] D. W. R. Balkenende , S. Coulibaly , S. Balog , Y. C. Simon , G. L. Fiore , C. Weder , J. Am. Chem. Soc. 2014, 136, 10493–10498. 24972163 10.1021/ja5051633

[5] C.-H. Li , C. Wang , C. Keplinger , J.-L. Zuo , L. Jin , Y. Sun , P. Zheng , Y. Cao , F. Lissel , C. Linder , X.-Z. You , Z. Bao , Nat. Chem. 2016, 8, 618. 27219708 10.1038/nchem.2492

[6] M. Burnworth , L. Tang , J. R. Kumpfer , A. J. Duncan , F. L. Beyer , G. L. Fiore , S. J. Rowan , C. Weder , Nature 2011, 472, 334. 21512571 10.1038/nature09963

[7] H. Zhou , M. Chen , Y. Liu , S. Wu , Macromol. Rapid Commun. 2018, 39, 1800372. 10.1002/marc.201800372 30091799

[8] J. Steinkoenig , M. M. Zieger , H. Mutlu , C. Barner-Kowollik , Macromolecules 2017, 50, 5385–5391.

[9] P. Klán , T. Solomek , C. G. Bochet , A. Blanc , R. Givens , M. Rubina , V. Popik , A. Kostikov , J. Wirz , Chem. Rev. 2013, 113, 119–191. 23256727 10.1021/cr300177k PMC3557858

[10a] M. A. Azagarsamy , D. D. McKinnon , D. L. Alge , K. S. Anseth , ACS Macro Lett. 2014, 3, 515–519; 10.1021/mz500230p 35590721

[10b] M. W. Tibbitt , A. M. Kloxin , K. S. Anseth , J. Polym. Sci. Part A 2013, 51, 1899–1911. 10.1002/pola.26574 PMC3785226 24496479

[11a] M. Lunzer , L. Shi , O. G. Andriotis , P. Gruber , M. Markovic , P. J. Thurner , D. Ossipov , R. Liska , A. Ovsianikov , Angew. Chem. Int. Ed. 2018, 57, 15122–15127; 10.1002/anie.201808908 PMC6391948 30191643

[11b] H. Zhao , E. S. Sterner , E. B. Coughlin , P. Theato , Macromolecules 2012, 45, 1723–1736.

[12] A. Iturmendi , S. Theis , D. Maderegger , U. Monkowius , I. Teasdale , Macromol. Rapid Commun. 2018, 39, 1800377. 10.1002/marc.201800377 30048024

[13a] X.-H. Qin , X. Wang , M. Rottmar , B. J. Nelson , K. Maniura-Weber , Adv. Mater. 2018, 30, 1705564; 10.1002/adma.201705564 29333748

[13b] J. A. Peterson , C. Wijesooriya , E. J. Gehrmann , K. M. Mahoney , P. P. Goswami , T. R. Albright , A. Syed , A. S. Dutton , E. A. Smith , A. H. Winter , J. Am. Chem. Soc. 2018, 140, 7343–7346; 29775298 10.1021/jacs.8b04040

[13c] P. Xiao , J. Zhang , J. Zhao , M. H. Stenzel , Prog. Polym. Sci. 2017, 74, 1–33.

[14] J. T. Offenloch , M. Gernhard , J. P. Blinco , H. Frisch , H. Mutlu , C. Barner-Kowollik , Chem. Eur. J. 2019, 25, 3700–3709. 30238521 10.1002/chem.201803755

[15a] W. Sun , R. Thiramanas , L. D. Slep , X. Zeng , V. Mailänder , S. Wu , Chem. Eur. J. 2017, 23, 10832–10837; 28564102 10.1002/chem.201701224

[15b] W. Sun , M. Parowatkin , W. Steffen , H.-J. Butt , V. Mailänder , S. Wu , Adv. Healthc. Mater. 2016, 5, 467–473. 26680371 10.1002/adhm.201500827

[16a] V. San Miguel , M. Álvarez , O. Filevich , R. Etchenique , A. del Campo , Langmuir 2011, 27, 1217–1221; 10.1021/la2033687 22149173

[16b] M. Salierno , E. Marceca , D. S. Peterka , R. Yuste , R. Etchenique , J. Inorg. Biochem. 2010, 104, 418–422. 20060592 10.1016/j.jinorgbio.2009.12.004 PMC2835317

[17] S. Theis , A. Iturmendi , C. Gorsche , M. Orthofer , M. Lunzer , S. Baudis , A. Ovsianikov , R. Liska , U. Monkowius , I. Teasdale , Angew. Chem. Int. Ed. 2017, 56, 15857–15860; 10.1002/anie.201707321 PMC5725706 28941025

[18] T. L. Rapp , C. B. Highley , B. C. Manor , J. A. Burdick , I. J. Dmochowski , Chem. Eur. J. 2018, 24, 2328–2333. 29161461 10.1002/chem.201704580 PMC5915374

[19] O. Filevich , M. Salierno , R. Etchenique , J. Inorg. Biochem. 2010, 104, 1248–1251. 20825994 10.1016/j.jinorgbio.2010.08.003

[20] C. Gorsche , R. Harikrishna , S. Baudis , P. Knaack , B. Husar , J. Laeuger , H. Hoffmann , R. Liska , Anal. Chem. 2017, 89, 4958–4968. 28383904 10.1021/acs.analchem.7b00272

[21] P. M. Kharkar , K. L. Kiick , A. M. Kloxin , Polym. Chem. 2015, 6, 5565–5574. 26284125 10.1039/C5PY00750J PMC4536978

[22] L. Zayat , C. Calero , P. Alborés , L. Baraldo , R. Etchenique , J. Am. Chem. Soc. 2003, 125, 882–883. 12537482 10.1021/ja0278943

[23] K. Zeitler , Angew. Chem. Int. Ed. 2009, 48, 9785–9789; 10.1002/anie.200904056 19946918

[24a] R. Wollhofen , M. Axmann , P. Freudenthaler , C. Gabriel , C. Röhrl , H. Stangl , T. A. Klar , J. Jacak , ACS Appl. Mater. Interfaces 2018, 10, 1474–1479; 29280613 10.1021/acsami.7b13183 PMC5773935

[24b] R. Wollhofen , B. Buchegger , C. Eder , J. Jacak , J. Kreutzer , T. A. Klar , Opt. Mater. Express 2017, 7, 2538–2559;

[24c] B. Buchegger , J. Kreutzer , B. Plochberger , R. Wollhofen , D. Sivun , J. Jacak , G. J. Schütz , U. Schubert , T. A. Klar , ACS Nano 2016, 10, 1954–1959; 26816204 10.1021/acsnano.5b05863 PMC4768287

[24d] C. Wolfesberger , R. Wollhofen , B. Buchegger , J. Jacak , T. A. Klar , J. Nanobiotechnol. 2015, 13, 27; 10.1186/s12951-015-0084-6 PMC4453224 25888763

[24e] R. Wollhofen , J. Katzmann , C. Hrelescu , J. Jacak , T. A. Klar , Opt. Express 2013, 21, 10831–10840. 23669940 10.1364/OE.21.010831

[25] The multiphoton absorption for 1028 nm wavelength process, however, needs to be analyzed in more detail. The assumption of a multiphoton process is based on earlier findings, see, for example:

[25a] E. Fino , R. Araya , D. S. Peterka , M. Salierno , R. Etchenique , R. Yuste , Front. Neural Circuits 2009, 3, 1–9; 19506708 10.3389/neuro.04.002.2009 PMC2691658

[25b] M. Four , D. Riehl , O. Mongin , M. Blanchard-Desce , L. M. Lawson-Daku , J. Moreau , J. Chauvin , J. A. Delaire , G. Lemercier , Phys. Chem. Chem. Phys. 2011, 13, 17304–17312; 21879060 10.1039/c1cp21661a

[25c] B. J. Coe , M. Samoc , A. Samoc , L. Zhu , Y. Yi , Z. Shuai , J. Phys. Chem. A 2007, 111, 472–478. 17228896 10.1021/jp0656072

