# Supplementary material for: Dynamic Supramolecular Ruthenium‐Based Gels Responsive to Visible/NIR Light and Heat
Source: Chemistry. 2019 Jul 3;25(42):9851–5. doi: 10.1002/chem.201902088 (PMC6771519; doi:10.1002/chem.201902088)
Supplement: Supplementary file 1 — Supplementary [file CHEM-25-9851-s001.pdf]

CHEMISTRY
A European Journal

Supporting Information

Dynamic Supramolecular Ruthenium-Based Gels Responsive to Visible/NIR Light and Heat

Ian Teasdale,*[a] Sabrina Theis,[b] Aitziber Iturmendi,[a] Moritz Strobel,[c] Sabine Hild,[c] Jaroslaw Jacak,[d] Philipp Mayrhofer,[d] and Uwe Monkowius*[e]

chem_201902088_sm_miscellaneous_information.pdf

Materials and Methods All reagents were commercially obtained and used as received. Solvents were purchased from Merck and VWR. All other chemicals were purchased from Sigma Aldrich, Acros Organics and ABCR. $Ru(bpy)_2Cl_2$ was prepared according to the literature [1]. UV-vis spectra were recorded on a Cary 300 Bio photometer. The samples were irradiated with a HBO lamp with 100 W and a cut off filter of 395 nm (GG 395 nm from SCHOTT). The viscosity was measured with a rotary viscometer from Brookfield Digital-Viscometer, Modell DV-I+ Version 4.0 and the measurement body No. 6.

For rheometrical measurements the complex shear modulus was determined. This modulus represents the visco-elastic properties of the investigated sample. The applied shear strain and the measured shear stress show a certain phase lag δ: For an ideal viscous sample the phase lag would have a value of 90°, for an ideal elastic sample the phase lag would be 0°. Through determining the phase lag the complex shear modulus can be split up in the storage modulus G', which represents the elastic proportion of the complex shear modulus or stored energy of the sample, and the loss modulus G'', representing the viscous proportion of the complex shear modulus or the energy dissipated as heat.

The photo-rheology was performed on a MCR 502 Anton Paar Rheometer (Anton Paar GmbH, Graz, Austria). The samples were investigated by oscillatory rheology, with a plate-plate geometry attached. The samples were kept at 25°C by a temperature adjustable bottom plate. For measurement a 8 mm stamp, which was developed and provided by Anton Paar GmbH, was installed. To initiate the decomposition an UV-LED-laser was guided from the top through the rheometer stamp on the sample. The LVE-range was determined before measurements and the parameters were set to 1% deformation, a frequency of 10 rad s^{-1} and a gap size of 0.2 mm. As UV-light source a UV-LED smart (Opsytec Dr. Gröbel GmbH, Ettingen, Germany) was attached. The laser provided a parallel beam with an intensity of >25000 mW cm^{-2}, and a wavelength of 365 nm.

After decomposition in the rheometer, a sample was heated up to 50°C to reverse the process. The rise in the moduli after several minutes indicates the gelation process. The parameters of the rheometric measurements were the same as for the decomposition experiments. Due to a possible loss of the solvent upon heating, the absolute values are not reliable hence relative values are shown (see Figure S5).

Ruthenium-P4VP gel containg 5 mol% of Ru(bpy)$_2$Cl$_2$

Poly(4-vinylpyridine) (85.9 mg, 0.818 mmol -pyridine) was dissolved in 1 mL of methanol in the ultrasonic bath. After dissolution, Ru(bpy)$_2$Cl$_2$ (10.3 mg, 0.0213 mmol), suspended in 1.5 mL water, was added. The reaction mixture was heated at 80 °C for 16 hours and afterwards stirred for 1 hour at room temperature until complete gelation. In order to investigate the photodegradation, the gel was irradiated with visible light >395 nm. Upon irradiation the gel becomes liquid. To start a new cycle of gelation/de-gelation, the reaction mixture can be heated again for 16 hours at 80 °C. For gelation, the mixture was cooled to room temperature and stirred until gelation. The time which is necessary for gelation is referred to as “gelation time” and is a rough estimation to assess the changes of the gel in the course of the gelation/de-gelation cycles (Table S1). The time of irradiation which is necessary for de-gelation is referred to as “de-gelation time”.

Table S1. Approximate gelation/de-gelation times for the preparation of the ruthenium containing P4VP gel containing 5 mol% of Ru crosslinker.

**Number**	**Gelation time**	**De-gelation time upon irradiation**
**Cycle 1**	~60 min	~10 min
**Cycle 2**	~60 min	~10 min
**Cycle 3**	~60 min	~5 min
**Cycle 4**	3 days	~2 min
**Cycle 5**	5 days	~2 min

Ruthenium-P4VP gel containg 10 mol% of $Ru(bpy)_2Cl_2$

The gel was prepared using 84.2 mg (0.802 mmol -pyridine) of poly(4-vinylpyridine) and 19.4 mg (0.0401 mmol) of $Ru(bpy)_2Cl_2$. The parameters of the different cycles are summarized in **Fehler! Verweisquelle konnte nicht gefunden werden.**

Table S2. Parameters for the preparation of the ruthenium containing P4VP gel containing 10 mol% of crosslinker.

**Number**	**Gelation time**	**De-gelation time upon irradiation**
Cycle 1	~60 min	~30 min
Cycle 2	~60 min	~25 min
Cycle 3	~60 min	~15 min
Cycle 4	~60 min	~15 min
Cycle 5	~5 days	~5 min

Ruthenium-P4VP Gel with 15 mol% crosslinker

The gel was prepared using 86.8 mg (0.827 mmol -pyridine) of poly(4-vinylpyridine) and 30.4 mg (0.0628 mmol) of $Ru(bpy)_2Cl_2$. The parameters of the cycles are summarized in Table S3.

Table S3. Parameters for the preparation of the ruthenium containing P4VP gel containing 15 mol% of crosslinker.

Number	Gelation time	De-gelation time upon irradiation
Cycle 1	~30 min	~60 min
Cycle 2	~30 min	~40 min
Cycle 3	~30 min	~40 min
Cycle 4	~45 min	~30 min
Cycle 5	~45 min	~30 min
Cycle 6	~45 min	~30 min
Cycle 7	~45 min	~20 min
Cycle 8	~45 min	~20 min
Cycle 9	~60 min	~15 min
Cycle 10	~60 min	~15 min

Ruthenium-P4VP Gel with 20 mol% crosslinker

The gel was prepared using 89.3 mg (0.851 mmol -pyridine) of poly(4-vinylpyridine) and 41.4 mg (0.0855 mmol) of $Ru(bpy)_2Cl_2$.

Afterwards the reaction mixture was stirred for 15 minutes at room temperature until complete gelation. However, despite irradiation for more than 8 hours the consistency of the gel remained similar and no photoreaction took place.

Ruthenium-P4VP Gel for viscosity measurements

Poly(4-vinylpyridine) (705 mg, 6.714 mmol -pyridine) was dissolved in 8 mL of methanol in a ultrasonic bath. Afterwards $Ru(bpy)_2Cl_2$ (260 mg, 0.5372 mmol) suspended in 12 mL water was added. The reaction mixture was heated at 80 °C for 16 hours and then stirred for 30 minutes at room temperature while forming a gel. This gel was irradiated for 3 hours until the reaction mixture was liquid and the cycle started from the beginning.

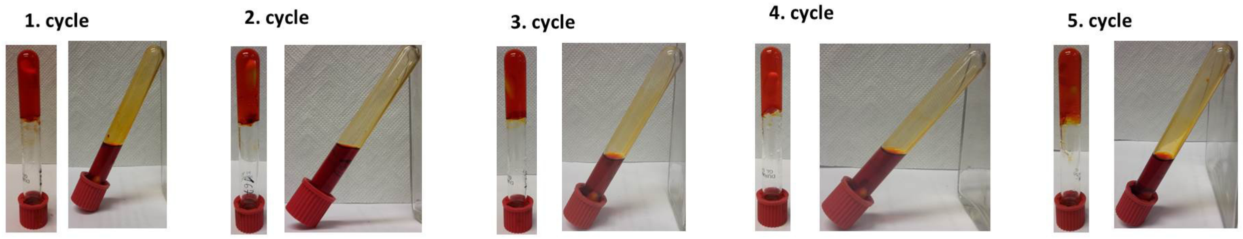

Figure S1. Gelation/de-gelation cycles of the gel containing 5 mol% of $Ru(bpy)_2Cl_2$ crosslinker. Irradiation with visible light > 395 nm.

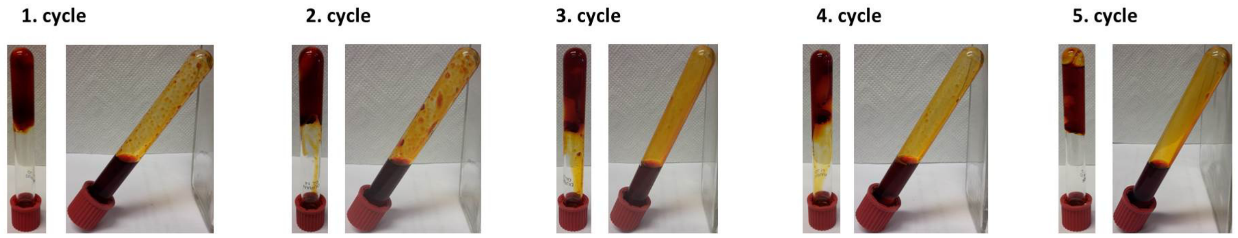

Figure S2. Gelation/de-gelation cycles of the gel containing 10 mol% of $Ru(bpy)_2Cl_2$ crosslinker. Irradiation with visible light > 395 nm.

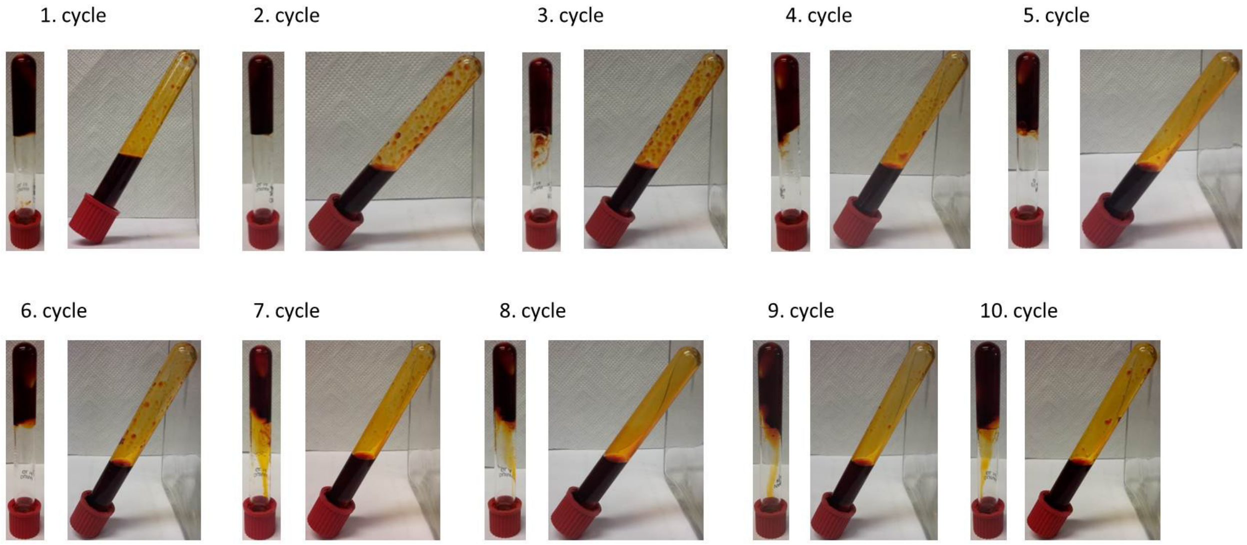

Figure S3. Gelation/de-gelation cycles of the gel containing 15 mol% of $Ru(bpy)_2Cl_2$ crosslinker. Irradiation with visible light > 395 nm.

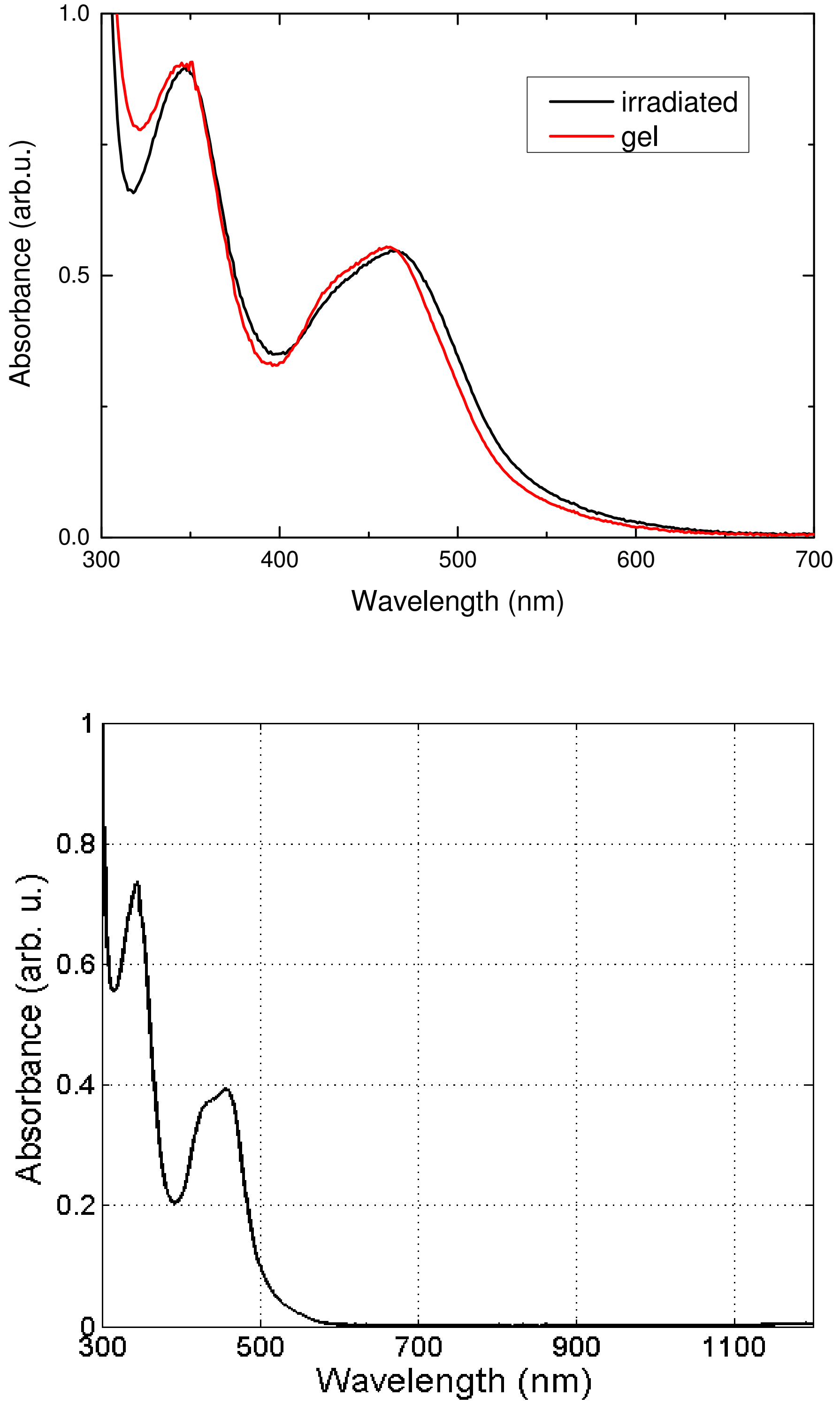

Figure S4. Absorption spectra of the 5%-gel and the irradiated liquid (top) and extended absorption spectrum of the 5% gel proving that there is no considerable absorption up to 1200 nm (bottom).

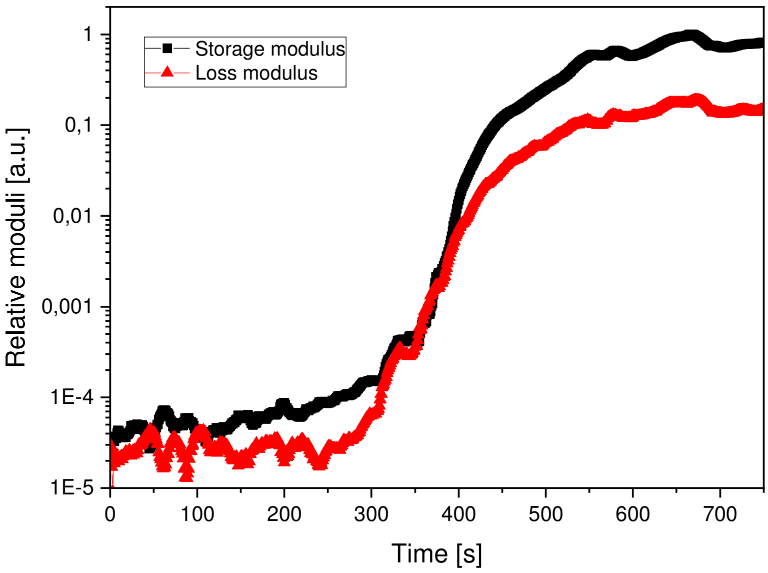

Figure S5. Rise of the rheometric moduli at 50°C indicating the gelation

Experiment for rotary viscometry (15%)

Poly(4-vinylpyridine) (705 mg) was dissolved in 8 mL of methanol in the ultrasonic bath. After dissolution, $Ru(bpy)_2Cl_2$ (260 mg) suspended in 12 mL water was added. The reaction mixture was heated at 80 °C for 16 hours. Afterwards the solution was stirred for 30 minutes at room temperature while forming a gel. The gel was irradiated for 3 hours until the reaction mixture was liquid. This cycle was repeated for 8 times.

The viscosity was measured with a rotational viscometer from Brookfield Digital-Viscometer, Modell DV-I+ Version 4.0 and the measurement body No. 6.

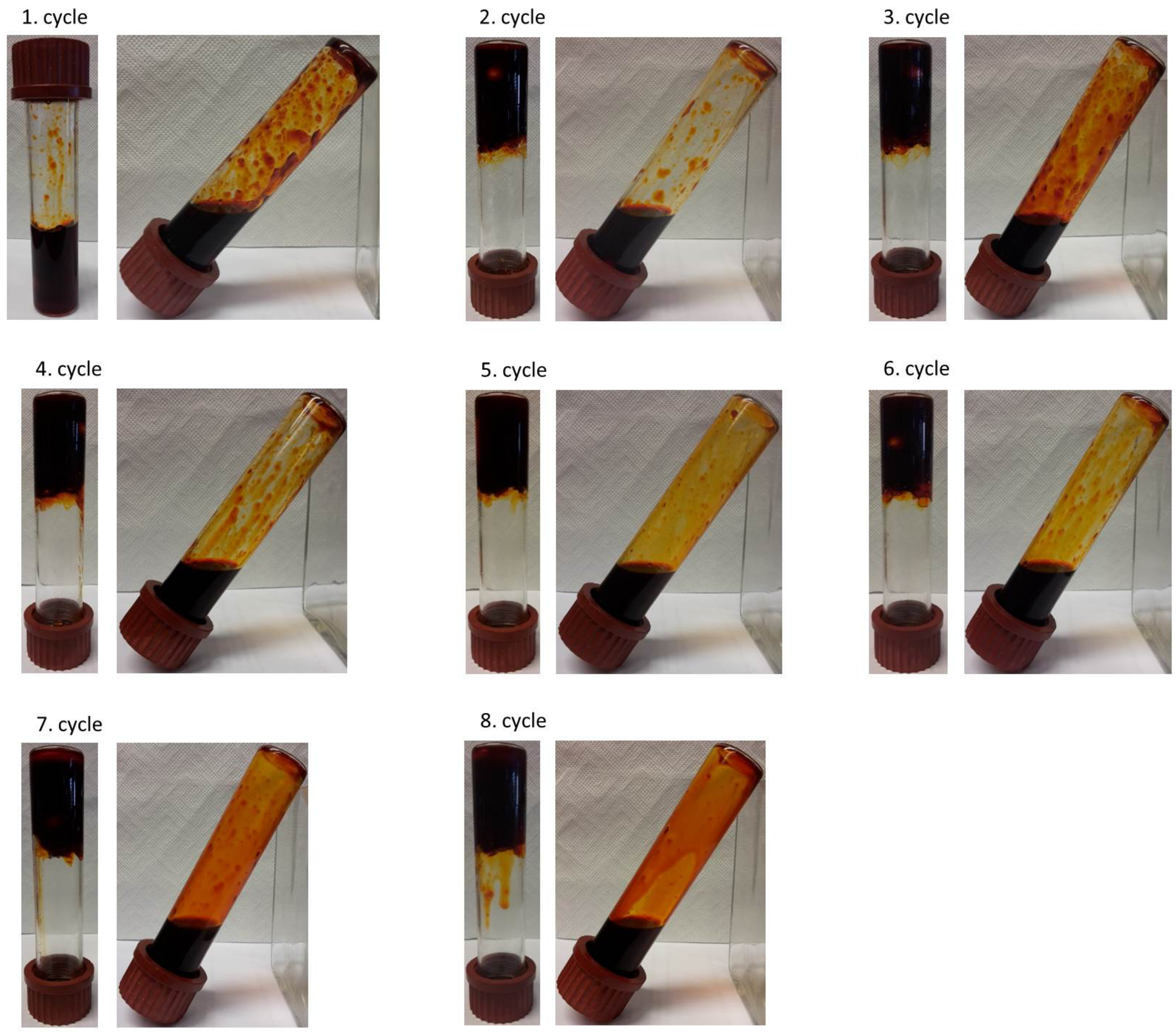

Figure S6. Gelation/de-gelation cycles of the gel containing 15 mol% of $Ru(bpy)_2Cl_2$ crosslinker. Irradiation with visible light > 395 nm.

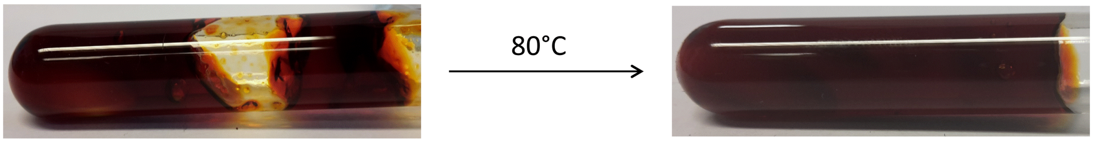

Figure S7. The area in the middle was irradiated with visible light > 395 nm. By heating the reaction mixture to 80 °C the gel has the ability for self-healing and an uniform gel was obtained again.

Lithography

For imaging an infinity corrected NIR air microscopy objective (Mitutoyo, M Plan Apo NIR 50x/0.42, Japan) and an industrial camera (UI-5240CP, Imaging Development Systems) are used.

Pictures G-I (Figure S8) are acquired by a single photon counting unit (PDM, Micro Photon Devices) using the same air objective.

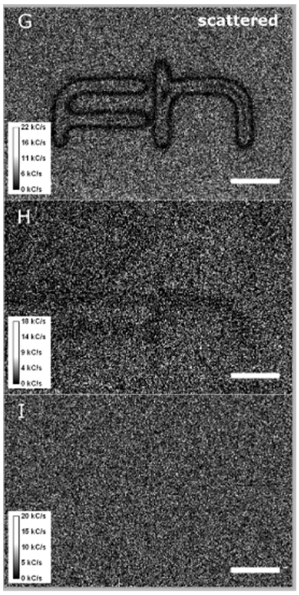

Figure S8. Backscattered image: All scale bars are 50 µm. G) shows the contrasted backscattered light image after MPL structured logo in the 5wt% hydrogel. H) Backscattered light image of the same area after a short curing process. The logo has partially disappeared from the image. I) Backscattered light image of the same area after finished curing process.

---

[1] B.P. Sullivan, D. J. Salmon, T. J. Meyer, Mixed phosphine 2,2'-bipyridine complexes of ruthenium, *Inorg. Chem.* **1978**, *17*, 3334-3341.
